# Has Menstruation Disappeared? Functional Hypothalamic Amenorrhea—What Is This Story about?

**DOI:** 10.3390/nu13082827

**Published:** 2021-08-17

**Authors:** Karina Ryterska, Agnieszka Kordek, Patrycja Załęska

**Affiliations:** 1 Department of Human Nutrition and Metabolomics, Pomeranian Medical University in Szczecin, 70-204 Szczecin, Poland; patrycjaazaleska288@gmail.com; 2 Neonatal Clinic, Pomeranian Medical University in Szczecin, 70-204 Szczecin, Poland; agkordek@pum.edu.pl

**Keywords:** gynecologic disease, amenorrhea, nutrition, ovary, uterus, stress, low energy, physical activity

## Abstract

Functional hypothalamic amenorrhea (FHA) is a very common condition affecting women of procreative age. There are many reasons for this disorder, including a low availability of energy in the diet, low micro- and macronutrient intake, overly intensive physical activity, disturbed regeneration processes, sleep disorders, stress, and psychological disorders. The main determinant is long-term stress and an inability to handle the effects of that stress. FHA is a very complex disorder and often goes undiagnosed. Moreover, therapeutic interventions do not address all the causes of the disorder, which could have implications for women’s health. As shown by scientific reports, this condition can be reversed by modifying its causes. This review of the literature aims to update the current knowledge of functional hypothalamic amenorrhea and underscores the complexity of the disorder, with particular emphasis on the nutritional aspects and potential interventions for restoring balance.

## 1. Introduction

Functional hypothalamic amenorrhea (FHA) is one of the most common menstruation disorders among women of childbearing age. The diagnosis of FHA is based on the exclusion of other causes of non-menstruation, including organic and anatomical factors [1,2,3,4]. This status should be reversible upon the modification of the basic causes.

The main determinant of the disorder is a combination of psychosocial and metabolic stress. Predisposing factors include low energy availability [1], nutrient deficiencies [1,5], excessive physical activity [1,2], a lack of endometrium regeneration [1,2], abnormal sleep [1,2], emotional tension [1,2], unmanageable chronic or severe stress [1,2], and dysfunctional behavior [1,2,5]. A common occurrence in FHA is the co-existence of many components. Previous reports suggested the crucial importance of body weight in the pathogenesis of the disorder. However, it is now clear that functional menstruation disorders are diagnosed in people with wide range of body weight and body fat. Neuroendocrine aberrations may also occur despite a normal body weight [4,5,6].

In response to the above mentioned factors, the pulsatory secretion of gonadotropin releasing hormone (GnRH) in the hypothalamus is blocked, which results in the abnormal secretion of tropic hormones by the pituitary gland, including follicotropins (FSH) and lutropin (LH) [1,3,7,8]. As a consequence, estrogen production is reduced, and no ovulation occurs. Progesterone is also absent since ovulation is completely blocked. Progesterone is produced by the luteinization of granulosa cells of the ovulating follicle. The entire monthly cycle is deregulated and, over time, becomes completely absent [1,3,7,8].

**Figure 1:**
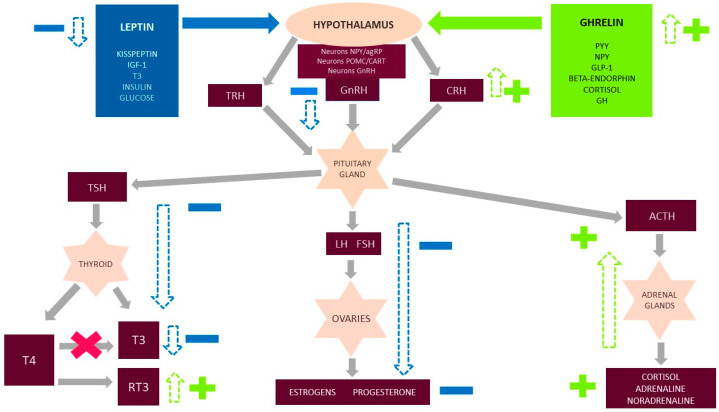
Functional hypothalamic amenorrhea (FHA) and influence on the endocrine system. Figure 1 shows the major hormone axis dysfunction caused by FHA, including the additional important neurohormonal factors that decrease (leptin, kisspeptin, IGF-1, FT3, insulin, and glucose) and increase (ghrelin and peptide YY (PYY), neuropeptide Y (NPY), growth hormone (GH), glucagon-like peptide 1 (GLP-1), beta-endorphins, and cortisol) in FHA. The hypothalamus secretes gonadotropin releasing hormone (GnRH), corticotropin-releasing hormone (CRH), and thyrotropin-releasing hormone (TRH), which then affect the pituitary gland and stimulate it to secrete tropic hormones: lutropin (LH), follicotropins (FSH), adrenocorticotropic hormone (ACTH), and thyroid-stimulating hormone (TSH). These hormones then affect the target organs. In turn, the ovaries, adrenal glands, and thyroid glands secrete hormones specific to them. Under physiological conditions, all hormonal axes are regulated by positive and negative feedback. A decreased concentration of hormones occurs in each of the secretory stages for the hypothalamus–pituitary–ovary (HPO) and hypothalamus–pituitary–thyroid (HPT) axes. Moreover, in the case of the thyroid gland, the conversion of T4 to the metabolically active form of T3 is disturbed. Instead, the inactive form reverse-T3 is produced in excess and blocks the receptors for T3. As a result of metabolic adaptation, for protective purposes, the low levels of gonadal and thyroid hormones in FHA do not stimulate positive feedback. The HPA axis is also overstimulated at each stage of regulation. As a consequence, there is an increased level of cortisol, which may not have an inhibitory effect with negative feedback at the higher centers of axis regulation.

Although the onomastics suggest a disorder associated with reproductive functions, FHA is closely correlated with the regulation of the entire endocrine system and the neurotransmitter system. The following metabolic and psychological consequences have been described (Figure 1) [7]: The hypothalamus–pituitary–ovary axis ceases to function, and the hypothalamus and pituitary and thyroid glands are affected. These consequences manifest as a reduction in relevant activities. The TSH level decreases or lies at the lower limit of the standard range, as does the T4 level. The T3 concentration is also reduced, and the conversion of T4 to the active metabolite T3 is impaired. T4 is converted into a non-active reverse-T3 that blocks T3 receptors [1,3]. In response to chronic stress, the HPA axis is enhanced at each step of regulation, which results in chronically elevated cortisol levels and subtly regulated rhythms over about 24 h. A reduced concentration of leptin plays a significant role in this process. The characteristic results include reduced glucose, insulin, insulin-like growth factor (IGF-1), and kisspeptin along with elevated ghrelin, growth hormone (GH), Y neuropeptide Y, peptide YY, and beta-endorphin [3,5,6,7,8,9]. Kisspeptin, encoded by the Kiss-1 gene, is a hormone produced in the hypothalamus that plays a key role in the direct stimulation and release of GnRH [1]. Moreover, some studies have suggested a positive correlation between kisspeptin and LH secretory pulses [10]. Kisspeptin may also influence the negative and positive feedback of estrogen [11]. Many reports have emphasized the sensitivity of kisspeptin to the metabolic state of the body and to stress, both acute and chronic [10,11]. It was reported that the higher the cortisol level is, the lower the plasma level of kisspeptin will be [10]. Intriguing observations were described in one study, which observed that the subcutaneous injection of kisspeptin in women with FHA caused the secretion of gonadotropins and an increase in estradiol concentrations [10].

Notably, changes in menstruation are the latter signs of the disorder. Symptoms such as a lack of ovulation and abridged luteal phase are of primary concern [1,12]. These symptoms occur despite normal monthly bleeding. Without the use of specialized tests, these problems often go unnoticed. Other, more pronounced signals include irregular menstruation, elongated cycles, and spotting, referred to as “oligo-polymenorrhea”. The last step is the complete lack of menses. For women who regularly menstruate, menstrual atrophy can occur more than 3 months. On the other hand, in the case of irregular cycles, menstrual atrophy can occur over 6 months [1,7,12].

In light of past scientific reports, it can be concluded that multidirectional operations are needed. Based primarily on a combination of work with the patient’s psyche and primary improvement in nutritional status. Consistent activities should include a modification of eating habits and training [13,14,15].

## 2. Pathogenicity

### 2.1. Psychology

In the literature, the psychological profile of women with functional menstruation disorders is quite well characterized. This profile includes perfectionism [16,17], high demands for oneself and others [14], low self-esteem [16], introversion [16], a fear of judgment [16], a strong need for social acceptance [16], problems with communication and social networking [16], a fear of maturity and sexuality, an inability to deal with daily stress and problems [1,16,17,18], and an inability to define one’s emotions [1,16]. In addition, high levels of anxiety [14], depressed mood [14,16], depression [14,16], and sleep disorders have been noted [1,14,15,16,17,18].

Abnormal nutrition and physical activity, an increased focus on diet, and a fear of gaining weight are also common [6,18,19]. These symptoms have been observed despite the elimination of women with clinical nutrition disorders from the sample groups [13,17,20]. Some reports, moreover, suggest that FHA is a milder form of ED (eating disorders) [16], with a much lesser degree of mental disorders and cognitive impairment but even more easily altered thinking and conduct [4]. However, it is important to remain vigilant, as eating disorders can often assume different forms that are not always simple to identify. The first step should always be to consult a doctor and a therapist [13,16,21,22]. The characteristics and attitudes mentioned above can have a very strong influence on the patient’s disturbed perception of reality and increased sensitivity to stressors [14,16,17,23,24]. In addition, the inability to modify cognitive patterns, engage in appropriate activities, and reduce stress can increase tension and anxiety. It was also underscored that individual susceptibility to stress is important [14,23], and FHA is most likely to increase through natural means. Some studies suggest that this sensitivity may be genetic [16]. It would be interesting to determine if there is a phenotype forming a system of neurotransmitters that lead to FHA [13,14,16].

In addition, scientific reports show that women’s exposure to stress during the prenatal period significantly affects the unborn child and results in increased sensitivity to the HPA axis. Early childhood experiences have similar effects [14,16]. In combination with education, predisposition can significantly contribute to shaping irregular attitudes and problems in life. In addition to cognitive dysfunctions, the stress related to everyday life and an inability to deal with that stress, abnormal sleep patterns, excessive physical activity, and a scarcity of energy from nutrients combine to form a “snowball” mechanism and enhanced reactivity [14,17,18,23,25,26].

Thus, on the one hand, the above characteristics are common in FHA. On the other hand, an imbalance of neurotransmitters, the overstimulation of the HPA axis, elevated cortisol levels, thyroid suppression, and reduced estrogen levels result in an increased occurrence of depressive states and strengthen the characteristics of the disorder [9,25,26].

A similar mechanism affects sleep. Excessive excitation of the sympathetic nervous system, chronic stress, anxiety, and tension are all conducive to sleeping disorders. Moreover, the lack of optimal quantitative and qualitative regeneration at night according to circadian rhythms has numerous health implications, both physical and mental. These implications have a strong negative impact on the whole endocrine economy, especially cortisol and leptin. In addition, a lack of adequate regeneration increases the risk of depression and contributes to greater irritability, deterioration, and decreases in cognitive function and efficient responses. A lack of adequate regeneration also weakens resilience to stress and available psycho-energy resources, thereby making a smaller stimulus produce a much greater effect [27,28].

### 2.2. Nutrition

In a situation of energy scarcity, the body minimizes its energy expenditures by curtailing less-important functions that are not essential for survival, such as menstruation. Survival becomes the overriding goal in increasing the activation of the sympathetic nervous system during a crisis. The menstrual cycle is an energy-intensive process. After pregnancy, lactation increases the expenditures even more. Caring for two organisms simultaneously then becomes impossible due to a lack of energy, components, and resources for the mother herself. The brain interprets energy scarcity and stress as adverse environmental conditions for the birth of the offspring. All these factors impede reproductive functions and offer protection for the body of the woman and her child. In the literature, this state is described as a reproductive compromise [6,7,8,18,29,30].

A well-known important determinant for FHA development is low energy availability. This issue is due to a lack of adaptation to the needs of food consumption and/or increased energy expenditures linked to physical activity. Both components frequently occur together. Regardless of the substrate, this condition generates a deficit of energy that translates into metabolic stress on the body. Nutrient deficiencies can be an additional aggravating factor (including of basic macronutrients, minerals, and vitamins) [5,15,20,31,32,33].

Energy is defined as the energy pool that remains for the body to use to maintain homeostasis, proper functioning, and optimal health. Energy availability (EA) is calculated based on the quantity of calories supplied with the diet after deducting the energy expenditures associated with the training divided by the non-fatty mass of the body [6,8,19,30].

Formula: EA = energy supplied from diet (kcal) − energy expenditure during training (kcal)/non-fat body weight (kg).(1)

This is the most widely cited pattern in the FHA literature alongside low energy availability. Because of its simplicity, this pattern is practical to enumerate [6,30,34]. Unlike classic energy balance, where the measurement is estimated for the total weight of the body, for the EA model in this template, the value of energy is determined in relation to the non-fat body weight. This type of body mass is significantly more active and generates a higher energy cost [35]. In addition, lean body mass (LBM) is generally higher in active individuals, suggesting that this parameter is more accurate. However, this parameter cannot be regarded as universally precise, reliable, or adequate. The estimated demand requires confirming other patterns and assessing the practical applications for each patient. Regardless of the formula used, the calculation of energy consumption expenditures and daily activity alongside controlling food intake under standard conditions may be a mistake and cause a great deal of difficulties [5,36]. Such measures must be taken with a high degree of caution in planning and monitoring [30,32,33].

**Figure 2:**
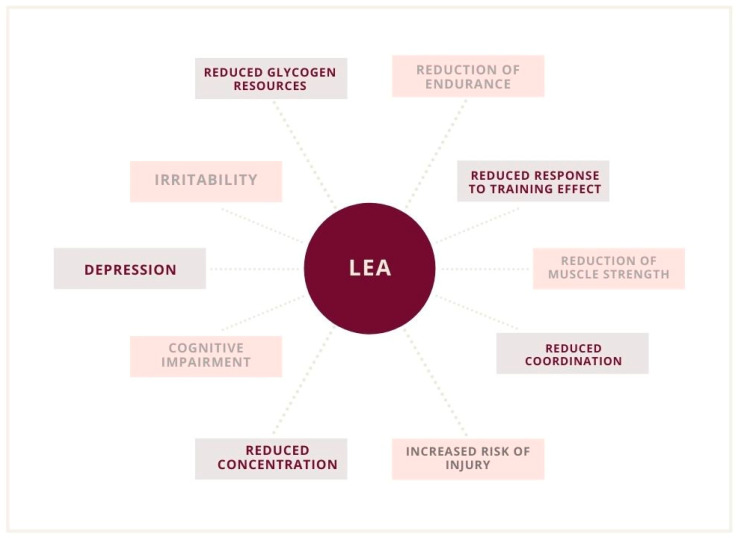
Psychological and physiological implications of low energy availability (LEA).

**Figure 3:**
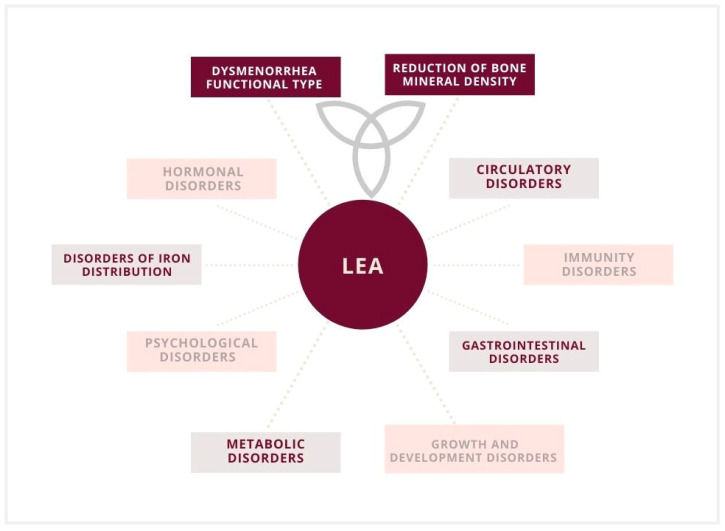
Low energy availability (LEA) and implications in a female athlete triad, including the functional type of dysmenorrhea and a reduction in bone mineral density.

A 2003 study suggested a threshold of 30 kcal/kg to initiate the disorder [37]. However, in subsequent studies, irregularities were observed in broad ranges, even above the threshold of 30 kcal/kg LBM. Some scientists also observed functional disturbances even when the declared EA was at an optimum level. However, changes in macronutrient consumption and other variables that could contribute to latent low energy availability (LEA) were also recognized [20,38]. However, it was not certain that the patient’s reported measurements were correct. Ultimately, with an increase in the deficit, the severity of health consequences also increases [12,30]. Undoubtedly, low energy availability is associated with a serious threat to the organism, which is significant for reproduction functions (Figure 2 and Figure 3) [7,8,19,32].

It is presently difficult to estimate the exact energy limits that could contribute to menstrual disorders. This is likely due to a number of factors contributing to energy expenditure and consumption. Each patient’s individual sensitivity, which has been consistently emphasized by researchers, also plays a key role [14,18,29,30].

However, there are certain thresholds in the literature that can be used as indicators for measurements. Low EA is determined as ≤ 30 kcal (125 kJ)/1 kg Fat Free Mass (FFM) per day. This value is similar to the basal metabolic rate [6,30]. The reduced energy availability risk shall be expressed in the range of 30–45 kcal (125–188 kJ)/1 kg FFM per day. This range is considered to entail a risk of reduced energy availability and is thus recommended only for a short time to reduce body fat. The correct EA is defined as ≥ 45 kcal (188 kJ)/1 kg FFM per day. Previous studies noted that this formula provides a ceiling close to “zero” for the energy balance [30,32]. In the event of pre-existing disorders, this value is likely to be necessary to restore lost menstruation and ovulation. The amount of energy spent on scheduled physical activity should also be added to this scheme [6,8].

LEA plays a significant role in the food consumption of FHA women and seems to impact not only the amount of energy delivered but also the distribution of individual macronutrients [5,32]. A lower intake of fat and carbohydrates was recognized in studies monitoring food intake among the sample group [20,39]. Easily digestible carbohydrates are easily accessible, and the supply of dietary fiber and low energy density products is generally high [20]. While the amount of protein supplied is debatable, the protein quantity usually falls within the upper limit of the standard or is even above [20,31,32,33]. These conclusions indicate the specific and interesting features of eating habits. On the one hand, these habits may result from modern trends in nutrition that do not necessarily coincide with the principles of proper nutrition. On the other hand, some diets correlate with eating disorders or abnormal attitudes. It is also possible to lack sufficient knowledge and mistakenly believe that the consumption of a diet with more fiber, protein, and food with low energy density is beneficial to the health of a physically active person. Each macronutrient has an individualized role in the body, which is also crucial in the context of menstrual function and energy availability. Some studies suggest that an increased participation of dietary fiber and dietary protein could contribute to a widening energy deficit despite the delivery of an optimal amount of energy [20]. Considering the characteristics of both components, fiber and protein, the influence between the two may be multidirectional. It is important for one to ensure an optimal supply of fat and carbohydrates in the diet to support the endocrine system.

Studies increasingly highlight the importance of the continuous availability of readily oxidized fuels [6,21,30,38]. The justification for such fuels is, above all, the sensitivity of LH pulsation to glycogen resources. A previous study noted that short-term deficits in women potentially not at risk affected the luteal phase, which is one of the first symptoms of menstrual abnormalities [12]. Another important nutritional factor is ensuring an appropriate amount of carbohydrates in the diet, as carbohydrates are the primary and fastest energy source for an organism and thus an indicator of energy balance [20]. A low supply of carbohydrates is correlated with the depletion of glycogen, leading to glycogen depletion [38].

Fat, which is a basic feedstock for steroid hormone synthesis, tends to be an insignificant component in the diets of women with menstrual disorders. At the same time, fat intake appears to be essential to ensure that omega-family fatty acids are present in the appropriate concentrations to reduce inflammation in the body [18,22]. For women with FHA, it is worth highlighting the role of omega-3 fatty acids in reducing inflammation associated with a spectrum of interactions [18,22,40]. There are indications that these fatty acids may improve menstrual-cycle-related ailments, as well as fertility [41,42]. Omega-3 fatty acids have also been shown to reduce perceived stress and anxiety in PMS and menopause, which are both states in which the amount of sex hormones is low, as in FHA [41]. Previous research also suggested that omega-3 fatty acids can support the prevention of depression and exert beneficial effects on the cardiovascular system and lipid regulation [40,43,44]—All of which could be affected to a large extent in the women in the sample group [1,9,45].

Studies have also suggested that not only the total amount of calories delivered but also the caloric distribution throughout the day is important for normal hormone pulsation [6], in order to avoid periods of deficiency, which can be translated into hourly deficits in energy availability. These values were correlated with higher cortisol levels and lower levels of T3 and sex hormones [38,46], particularly during the training period, which could significantly increase the exhaustion of resources [6]. The regular distribution of meals during the day and the avoidance of periods of hunger are basic tasks in diet planning.

Apart from the supply of energy and macronutrients, dietary micronutrients and vitamins play an important role in FHA. Vitamin D3 is a significantly deficient component in the general population and is difficult to supplement with one’s daily diet [47]. This disorder is exacerbated among the group of women at risk of FHA [9,45,47]. Vitamin D3 has many functions in the body and is one of the key factors involved in the body’s skeletal economy. Bone mineral density is extremely sensitive to functional endocrine disruptors [1,9,22]. In addition, there is evidence that bone mineral density can have a positive impact on mood and cognitive function, countering depression, which may provide considerable support for the target group of women [48]. Vitamin D3 may reduce inflammation and hepcidin levels, thereby increasing the absorption of iron [49]. Some studies have suggested that vitamin D deficiency may be associated with impaired fertility, but more evidence is needed. In addition, a correlation was observed between the loss of this component, lengthening of the follicular phase, and reduction of the luteal phase [50].

Calcium has many important functions in the body and is one of the key players in the context of bone mineral density [1,51]. Bone-related disorders are significantly more prevalent in this population of women [9,45,49].

Magnesium has been well-studied in the context of stress, anxiety disorders, and depression [47,51], which are often observed in women with FHA. In the case of magnesium, a vicious cycle was observed [52]. There is evidence that, in response to acute and chronic stress, magnesium resources are depleted, and the urinary output of magnesium is increased. Stressors may have a variety of backgrounds, both psychological and environmental. Sleep deprivation and intense physical exertion are also important [51,52], as is a lack of energy. Lack of energy is the main reason for increases in nutrient deficits. However, a scarcity of magnesium can amplify the symptoms of and susceptibility to both stress and depression [52]. In addition, magnesium performs many important functions in the body and is a co-factor in over 300 enzymatic reactions [52]. There are also reports that magnesium can improve the metabolism of vitamin D3 [53].

Monthly bleeding is not present, or is very rare and mild, in the studied group of women. Thus, such women do not experience increased monthly losses of iron resources. Nevertheless, deficits of iron [22,54] can be observed as the first factor, possibly due to insufficient consumption of iron in the diet and/or the presence of ingredients that limit the absorption of this element, such as excess fiber and phytic acid [17.56]. The second most important factor is increased physical activity, particularly in endurance tests [49]. The third factor is an inflammatory state that contributes to the production of hepcidin, a hormone that blocks the absorption of iron from the gastrointestinal tract. In addition, a deficiency of this element can significantly increase apathy and mood swings and decrease lactation [49].

Folate appears to be an essential component in the normal development and preparation of pregnancy. However, regardless of whether fertilization is a desirable outcome, this nutrient should be adequately supplied in the diet. There are some indications that folates can have a beneficial effect on menstrual cycle regulation and ovulation [41,55,56]. It was suggested that this phenomenon may be related to homocysteine [41,55]. Folate deficiency can contribute to the hypomethylation of DNA and oxidative stress [55]. Previous studies suggested that, in the case of mutations in the MTHR C677T gene and T-allele carriers associated with lower enzyme activity, a lower sensitivity of oocytes to the FSH hormone, reduced oocytes, and reduced estradiol production by granular cells compared to the case with vectors of the wild-type gene can occur [41,56,57]. In addition, folates can support vascular endothelial functions. Women with FHA commonly present folate dysfunctions [1,6,9,19,56].

It was long thought that the most critical factor in FHA development was insufficient body weight. However, the most important diagnostic parameter appears, instead, to be the composition of the body, specifically, fatty tissue content [5,15,31,58]. In athletes, it is recognized that, as a result of the development of greater-than-average muscle mass, the weight of the body can oscillate beyond the normal ranges. However, the athlete’s fat levels may be below the recommended minimum. The determination of clear standards is highly debatable and depends on individual factors [15]. However, if the above parameters and BMI are below the recommended standard, they must necessarily be restored to normal values [7,15,22,59]. It is commonly acknowledged that functional disorders can occur in those with broad ranges of both body weight and fat content, even when these parameters are correct and do not change over the years [1,4,5,6,29,32]. In addition, markers indicating low energy availability are also recognized in these women. Elevated levels of cortisol and ghrelin have been observed alongside reduced levels of sex hormones, T3, glucose, insulin, and leptin [5]. In many cases, the resting metabolic rate is also reduced. As a result of metabolic adaptation, the organism must minimize its expenditures and adapt [19,30,32,60]. Additional factors include psychosocial stress and physical activity, which can exacerbate stress on the body and impede reproduction [4,10,15].

### 2.3. Physical Activity

The greatest threat to FHA comes from forms of physical exercise in which aesthetics and the weight of the body play an important role, e.g., bodybuilding, dance, and gymnastics [6,15,19,22]. FHA is also common among amateurs [33,61,62,63], and women are becoming increasingly involved in physical activities. Unfortunately, without proper preparation, adaptation, and knowledge and the supervision of a specialist, there may be many risks.

In recent years, in the pathogenesis of functional menstruation disorders, sport has been primarily considered for inducing significant energy expenditures and compensating for energy consumption—sometimes due to ignorance and errors in the estimation of the exact components of the activity and sometimes due to an intentional, incorrectly designed, and prolonged fat-reduction program. Such issues may also be caused by the deliberate maintenance of a significant energy deficit, exacerbated by the stress of eating disorders or dysfunctional attitudes in these areas. An open and intriguing question is whether physical effort alone can result in hormonal deregulation. Most research discusses the generation of deficits. However, the fact that training itself is a stressor for the organism’s body should not be overlooked [2,59]. A systematic review considering the impacts of activity on ovulation noted that high-intensity activity affects the functioning of the reproductive system, particularly when one’s BMI is below the norm but also when the BMI is within a suitable range. Many mechanisms could be involved in this relationship [59]. Another study noted the inhibitory effect of exercise on sexual hormone concentrations. Interestingly, this effect was not dependent on energy availability. Attention has also been paid to the need to modify training volume [30].

Physical activity is often considered an excellent way to relieve tension and improve one’s mood. Unfortunately, for women with FHA, exercise could worsen their condition. Previous studies noted that, in response to a stress challenge, there is a significant increase in cortisol and a decrease in blood glucose compared to in healthy women [14,17,23,29]. This may indicate the significant depletion of energy resources and a mechanism to promote the organism’s mobilization to obtain such resources. This is an intriguing area of study, highlighting the need to consider the sensitivity of FHA women in relation to sporting activities.

Compulsive and physical dependence may also be problematic. These factors may lead to a situation where a person’s brain chemistry demands an increase in training load and frequency [13], despite that load being greater than what the individual can handle and/or inappropriate for particular circumstances [2,34,64,65,66]. This may lead to the phenomenon of over-training and related aberrations, and addiction and compulsion may lead to self-destruction in the mental, physical, and social fields. The characteristics and attitudes of FHA women were found to significantly increase dysfunction [8,34].

It should be noted that training alone is a stressor for an individual. Training stimulates the sympathetic nervous system and increases metabolic stress [2,29,37,58,59,67,68]. Training is often desirable for the development of an athlete. Regular physical activity is essential for staying healthy. The optimal dose and individual adjustment of the training parameters are crucial (they should be appropriate for the situation of the person undergoing training).

Stress and physical effort, in many cases, exacerbate the scarcity of resources and lead to an increase in demand. For amateurs, physical activity is only a supplementary part of life. Such individuals are burdened with many stressors resulting from everyday life, work, and school. Consequently, all components should co-exist, including a training plan, regeneration, and sleep; a diet that takes into account the consumption of not only a sufficient number of calories and macronutrients but also vitamins and minerals; and proper hydration.

## 3. Actionable Steps for Restoring Balance

Functional menstruation disorders can be characterized as psychosomatic diseases. Considering all the components, in many cases, it is necessary to simultaneously include multidirectional activities for each aspect so that changes can be smoothly implemented and to avoid prolonging the pathological state of the body [17]. Previous studies indicate that the longer the body’s decline persists, the longer the time needed to recover, and the more severe the consequences. Hence, time plays a significant role [22,54].

The first step should always be to consult an endocrine gynecologist and obtain a thorough diagnosis to exclude other diseases and control the current state of the body [1,2,69].

A detailed review of the patient’s diet, physical activity, feelings of stress, sleep, attitudes towards nutrition, and psychological profile is also crucial. Attention should be paid to whether the patient presents typical features of FHA and if an eating disorder is present. Sufficient data can help to locate the main cause of the problem, and primary causes the most destructive should be addressed first [1,23,24]. Endocrinological guidelines strongly recommend focusing on solving the behavioral issues that contributed to the problem. However, pharmacology is not recommended for first-line treatment because it only masks the return of natural menstruation due to ongoing or worsening undernourishment and exposure to stress [68]. In addition, hormone replacement therapy (HRT) does not affect the functioning of other hormones or improve bone mineral density if the dysfunctional condition is maintained [1,3,15,22].

Firstly, the nutritional status and eating habits of the individual should be assessed. In the vast majority of cases, an increase in energy consumption to the recommended value of 45 kcal/1 kg LBM supplies the amount of energy spent on training activities. The continuous observation of the patient’s response, however, remains important. The relevant calculations only provide an estimate, so they are not always entirely accurate [6,8,30,32]. In a study using a 360-kcal energy preparation without changes in sports activity for a period of six months, menstruation with ovulation was observed [39,70]. This study also observed a minimal increase 1.6 kg of body weight, which was adequate given the calories of the products used [39,70]. Another study used a 3-month intervention to increase energy supply and improve eating habits. As a result, 234 kcal of energy was generated, accompanied by an increase in the supply of macronutrients, vitamins, and minerals. Regular menstruation did not resume. However, increase of LH level and increase FSH to LH ratio level was observed and positively correlated with EA [61]. These parameters provide a promising indication of a gradual recovery in the balance and correct functions of the organism. The authors in [54] described cases of two different women who were physically active and had appropriate body weight and body-fat content. In response to an increase in energy availability, improvements in nutritional status markers (increases in T3 and leptin and a decrease in ghrelin) and the resumption of menstruation were noted. In both cases, however, the observed menstruation featured anovulatory cycles with the luteal phase. In addition, a re-stop was observed in the event of a drop in energy availability. Importantly, no target energy level was reached throughout the intervention. Consequently, the imposition of other factors and the lack of consistency could have contributed to the continuation of the problems of FHA [54].

Studies suggest that more attention should also be paid to the redistribution of energy over 24 h to avoid a latent deficit [38,46]. Dietary programming during the training period is particularly important. The regularity of one’s meals and a regular distribution according to one’s needs also appear to be beneficial. Too large of a gap between meals leads to significant drops in glucose; thus, fasting or intermittent fasting is not advisable. Based on studies analyzing the dietary habits of FHA women and their impact on the physiology and biochemistry of the organism, a key activity seems to be to ensure the balanced participation of macro components according to the recommended standards individually adapted to the patient’s situation [22].

Minerals and vitamins also play an important role. Special attention in the literature is given to vitamin D3 and calcium [1,22]. Studies also suggest the possibility of shortages of magnesium, zinc, iron, and folic acid; vitamins A, E, K, and C; and certain B vitamins [1,20,31,33]. The best solution is to address this shortage through one’s diet. It is, therefore, important to use high-nutrient-potential foods [22].

In the event of significant deficits or difficulties in meeting one’s dietary needs, targeted supplementation needs to be considered. In the study group, deficits in vitamins and minerals were a serious problem. On the one hand, excess stress, physical activity, and associated inflammation lead to increased expenditures of, and demands for, nutrients [51]. Moreover, low energy availability can impair basic metabolism, which in itself can exacerbate deficits [22]. In addition, an excess supply of dietary fiber can make it difficult to absorb certain nutrients [20], and an insufficiency of nutrient resources exerts metabolic stress on the organism and may hinder basic life processes [22,47,51]. Additionally, stress may negatively affect digestive enzymes and gut microbiota, which may result in the abnormal uptake of nutrients from the intake of food and/or their endogenous production [51].

Vitamin D3 is essential for the proper functioning of an organism but is difficult to acquire through food. It is thus recommended to include vitamin D3 supplements in one’s diet [48]. The dose should be selected following a previous examination of the blood concentration and tailored to each person’s individual needs. Another source of vitamin D3 is exposure to sunlight [48]. The concentration of calcium in the blood must be continuously monitored, and densitometry should also be performed in clinical settings [1]. The calcium availability in a patient’s diet should not be a problem, especially if the patient consumes dairy products. In addition, vitamin D3 supplementation should be considered to allow for a more efficient use of this element by the skeletal system [49]. Magnesium should also be easy to acquire through one’s diet. However, in the event of a magnesium deficit, it is worth considering supplementation. In particular, magnesium supplementation appears to be beneficial for women with menstrual disorders, not only those with functional disorders [71]. An adequate supply of magnesium can have a positive effect on a person’s mood, facilitate adaptation to stressful conditions, and reduce irritability. In addition, magnesium can benefit the initiation and quality of sleep [52]. In a previous study, exposure to severe mental and physical stressors, acute chronic stress, and physical effort were correlated with lower levels of zinc in the serum and plasma; zinc was also observed to have positive effects on the efficacy of antidepressants and lower cortisol levels [51]. Another intriguing observation was a reduction in inflammation and oxidative stress [72]. Iron supplementation can have a number of negative effects on one’s health. For example, excess iron can lower the concentration of leptin. The best solution seems to be an extended diagnosis and to address the shortage through one’s diet as far as possible. Supplementation should be used as a last resort and only under the ongoing supervision of a doctor. The best solution for securing a supply of folic acid appears to be its consumption within one’s diet because the folic acid in food products occurs in a methylated form. In the case of mutations such as those of the MTHFR C677T gene, synthetic folic acid supplementation is not effective. In this case, it is worth considering folic acid’s methylated form. Other factors involved in methylation should also be considered, such as vitamins B2, B6, and B12 and zinc [41,55]. Any decision should be made after consultation with a specialist and introduced on a case-by-case basis. The omega-3 fatty acids mentioned in the previous section play an important role in the functions of the body and have beneficial effects [40]. Determining the appropriate supply for one’s daily diet and the optimal balance between omega-3 and omega-6 fatty acids could, however, pose a significant challenge. The concentrations of individual omega-3 acids are also a key factor [40,43].

Only a few vitamins and minerals have been mentioned in this article. It is important to stress that each of the aforementioned nutrients is needed for the proper functioning of an organism, especially in the case of significant loads. B vitamins, especially folic acid, and antioxidants should not be neglected. To a large extent, the demand for nutrients allows a well-balanced diet to be achieved. It would also be beneficial to include recreational food in the diets of women with FHA (i.e., foods that are slightly more processed and have a higher energy density). Firstly, these foods will increase the quantity of calories delivered in a relatively simple way. Secondly, they could positively influence the psyches and satisfy the needs of FHA women. These measures would contribute to easing tension and preventing the use of drugs. The removal of such nutritional restrictions would help to improve nutritional relationships, encourage good eating habits, and facilitate healthy functioning [20].

A major factor to consider is the reduction of stressors. Physical activity, despite its many advantages, is one of these stressors. For women with FHA and malnutrition, its effect is compounded [14,23,29,58]. The issue of functional menstruation disorders is, moreover, common among athletes. Hence, a number of studies have focused on the above group. These researchers focused on using non-volatile training parameters. However, the modifications of those parameters were subject to energy consumption, and interventions produced varying results (also positive). This has led to the belief that physical activity can remain at a similar level over the long term. However, it is not appropriate to compare professionally trained trainers to amateurs with fewer training pressures, which could introduce further modifications. It is certainly not appropriate to generalize measures for applications requiring training units and the development of peak sport performance. Moreover, recreational athletes do not always have a professional training plan adapted to their current capabilities that reasonably accounts for the necessary regeneration periods. Studies suggest that excessive physical activity can occur among FHA women, which may be caused by eating disorders or other dysfunctions [13,34].

The complete avoidance of planned activities without the agreement of the patient does not appear to be an optimal solution. The avoidance of such activities may be stressful and generate anxiety when the sport is a daily routine, especially when it is related to eating disorders, compulsion, and/or addiction. In the above situation, it is important not to drive and support destructive behavior. On the other hand, it is worth considering modifications based on a compromise with the patient and coming to an agreement with solutions that are safe for that particular context. To minimize health risks, it should also be ensured that regeneration and nutritional status are maximized. Research further suggests the need to alter the training variables, such as by a reduction in the volume and intensity of the training [30,59]. It would also be beneficial to reduce the training frequency and introduce longer regeneration times, e.g., every other day. The types of training could also be altered. For example, yoga and related outdoor activities have a high potential to improve mental and physical health and do not generate increased excitability of the sympathetic nervous system; instead, they may have the opposite effect. However, resistance may be caused by patients’ anxiety in response to changing their exercise patterns, increasing their energy, and reducing their training parameters [6]. It is very important that experts make the patient aware of these issues. Patients should also work with a psychotherapist to reduce the relevant barriers and improve their psychological states [1,13,17,22]. Interventional studies on the use of psychotherapy in the treatment of FHA are already sufficient. The studies to date suggest including both cognitive and behavioral therapy due to promising results indicating the resumption of menstrual function with ovulation, increased leptin and T3 concentrations, and a decrease in ghrelin and cortisol levels [18,23,24]. Body weight also did not change, which is an additional positive aspect [18,23,24]. More research needs to be conducted in this area. However, given the low risk of undesirable activities, the pathogenesis of the problem, the characteristics of the group, and the benefits already examined, therapy appears to be an important element of recovery. This measure is not only likely to contribute to the correction of dysfunctional attitudes and thoughts but can also enable stress to be properly handled and diminished. In addition, nutrition and physical activity can be extremely beneficial. Ultimately, the benefits of psychotherapy seem to be significant in both psychosocial and physical terms [14,16,17,18,23,24].

## 4. Conclusions

Considering the overall pathogenesis of FHA, the most sensible approach is to combine improved nutritional status with physical activity and psychotherapy and work on daily stress. A broad range of relaxation techniques can complement these measures [14]. In addition, it is important to ensure that a patient obtains a good quality and quantity of sleep [27]. It is always important to bear in mind the individual sensitivity of the patient and maintain observations.

It is important to continuously and consistently maintain the initiated changes. The present study demonstrates that, the longer the time of decay, the more the time needed to regulate that decay. Changes, such as body weight reductions, are not recommended for 6 to 12 months after standardization. In addition, latent irregularities, such as a lack of ovulation and an abridged luteal phase, should also be prevented. This study indicates that the stressors occurring during the first phase of the cycle influence the delay of ovulation, leading to luteal dysfunction [30]. It is also important to ensure the continuous observation of the patient and a sensible approach both during and outside the recovery and maintenance period. Moreover, the individualization of interventions is crucial, and an interdisciplinary approach seems to be the best solution for promoting a promising prognosis.

## Data Availability

Not applicable.
